# Editorial: Demonstratives, Deictic Pointing and the Conceptualization of Space

**DOI:** 10.3389/fpsyg.2021.667667

**Published:** 2021-03-30

**Authors:** Holger Diessel, Kenny R. Coventry, Harmen B. Gudde, Olga Capirci

**Affiliations:** ^1^Department of English, Friedrich Schiller University Jena, Jena, Germany; ^2^School of Psychology, University of East Anglia, Norwich, United Kingdom; ^3^Institute of Cognitive Sciences and Technologies, Italian National Research Council, Rome, Italy

**Keywords:** deixis, demonstrative, pointing, gaze, joint attention

Demonstratives have been studied intensively across several disciplines. There is increasing evidence that demonstratives constitute a unique class of expressions, fundamentally distinct from all other types of linguistic items. In contrast to most other function words, demonstratives seem to exist in all languages and are not derived from content words (Diessel, 2013). Moreover, demonstratives are closely related to gestural communication, notably to pointing (Stukenbrock, 2015), and are among the first and most frequent words in early child language (Clark, 1978). Demonstratives also play an important role in the encoding of reference in space (Coventry et al., 2008) and the organization of linguistic elements in discourse (Himmelmann, 1997). Finally, demonstratives are crucially involved in the diachronic evolution of grammar, as many grammatical markers are historically based on demonstratives (Diessel, 2006). Taken together, these features characterize demonstratives as a very special class of linguistic items that are foundational to communication, spatial orientation, discourse processing, language acquisition, and the emergence of grammar.

This Research Topic includes 14 articles that provide an overview of current research on demonstratives and cast a fresh light on some of the above-mentioned topics. The articles are based on empirical and theoretical research from several disciplines and are concerned with various languages, including several signed languages and a tactile language. Most papers present original research using a variety of data and methods including data from comprehension and production experiments, electronic corpora, video recordings, mobile eye tracking glasses, parental reports of child language, field reports, and a typological database. In what follows, we briefly describe the 14 articles in this collection.

The first paper, by Diessel and Coventry, provides an interdisciplinary review of current research on demonstratives in linguistics and psychology. At center stage is the debate about the nature of demonstrative reference. Older studies analyzed demonstratives as a particular class of spatial terms, but in the recent literature it is often claimed that demonstratives are primarily used for social purposes. Diessel and Coventry argue that demonstratives have both spatial and social functions.

Talmy outlines a new theory of deixis and anaphora, which are traditionally often treated as separate phenomena. Challenging this view, Talmy argues that linguistic reference involves a single cognitive system, i.e., the targeting system, that includes both speech-external and speech-internal uses of demonstratives. The bulk of the paper explains how linguistic triggers for deictic and anaphoric reference are interpreted in light of different types of cues providing information about the referent and its location. Demonstrative pairs (e.g., *this/that)* are commonly used to contrast objects or spaces. In the Demonstrative Choice Task (DCT), developed by Rocca and Wallentin, demonstratives are paired with nouns in the absence of such contextual information. The DCT shows consistent effects of semantic features (i.e., manipulability, motion, time) on demonstrative choice, suggesting that demonstratives mark contrasts along a range of semantic as well as spatial dimensions.

The combination of demonstratives and pointing has been noted in numerous studies, but there is little research on demonstratives and gaze. The study by Stukenbrock uses a mobile eye tracking device to investigate the occurrence of gaze following in deictic communication. The study breaks new methodological ground by using this technology for the first time in order to examine multimodal uses of deictic reference in naturally occurring interactions.

In the acquisition literature, it is commonly assumed that demonstratives are among the earliest and most frequent words children use. Analyzing data from child language corpora and parental reports of English- and Spanish-speaking children, González-Peña et al. challenge this view. While their corpus data are not entirely conclusive, the data from parental reports suggest that demonstratives are not usually among the first 50 child words.

Using the Memory Game (Coventry et al., 2008; Gudde et al., 2018), Vulchanova et al. investigate the potential influence of a second language on the deictic system of speakers' native language. Comparing a group of monolingual Spanish speakers with a group of bilingual Spanish speakers learning Norwegian, they found a significant difference between groups, suggesting that the two-term system of Norwegian changed the way the bilinguals used the three-term system of their native language.

The paper by Forker is a cross-linguistic study on demonstratives encoding elevation (up vs. down). Contrary to what one might expect, the study shows that the occurrence of elevational demonstratives is not predictable from the topographic environment. Moreover, the paper shows that the encoding of elevation is often restricted to distal demonstratives and that elevational demonstratives can be mapped onto non-spatial domains, e.g., time.

Khachaturyan analyzes demonstratives in Mano, a Mande language of Papua New Guinea. Examining field data, the paper challenges the traditional distinction between endophoric and exophoric uses of demonstratives and argues that speakers' choice of a particular demonstrative is primarily determined by common ground rather than by type of reference.

A second field study is the article by Mesh et al., which is concerned with demonstratives in Quiahije Chatino, an indigenous language of Mexico. Specifically, this paper investigates the potential influence of the scale of the search space on the multimodal use of demonstratives. The study shows that speakers are more likely to combine demonstratives with a pointing gesture when the reference space involves a large scale rather than a local activity.

Reile et al. is an experimental study that investigates the influence of distance and salience on the alternation between proximal and distal terms in two different dialects of Estonian. There is good evidence that both factors influence speakers' choice of demonstratives; yet, there is an interesting difference between the two dialects, which the authors explain by the fact that in the northern variety demonstrative pronouns do not encode distance.

The final four papers are concerned with non-spoken languages. The article by Garcia and Sallandre, according to which anaphora is only one type of deixis, develops the theoretical framework known as the ‘Semiological Approach’ to analyse reference in sign language, recognizing the crucial importance of the role of gaze.

Wilcox and Martínez investigate the conceptualization of space in Argentine Sign Language based on video recordings. The authors show that space is used in various ways for creating reference in Argentine Sign language. Moreover, they argue, in accordance with Talmy, that deixis and anaphora form a continuum in signed languages that involves the same conceptual apparatus.

The paper by Cooperrider et al. examines how pointing is integrated into spoken and signed languages. Using a novel pointing elicitation task, these researchers found that both speakers and signers are especially likely to use a pointing gesture in conjunction with lexical expressions if the latter does not seem to be sufficient to identify the referent. Moreover, they observe that, while speakers tend to use points that span across words, signers' points typically occur in slots between lexical signs.

Finally, the paper by Edwards and Brentari is concerned with demonstratives in a tactile language, i.e., a language used by deafblind people. The analysis draws on video data and considers both synchronic and diachronic aspects of demonstratives. The diachronic data show that demonstratives have acquired new functions in a gradually emerging grammatical system, similar to the grammaticalization of demonstratives in spoken language.

In conclusion, this Research Topic provides a survey of current linguistic and psycholinguistic research on deixis and demonstratives yielding a number of new results that we hope will stimulate future research on this important topic.

## Author Contributions

All authors listed have made a substantial, direct and intellectual contribution to the work, and approved it for publication.

## Conflict of Interest

The authors declare that the research was conducted in the absence of any commercial or financial relationships that could be construed as a potential conflict of interest.
